# Parental Education and Aggressive Behavior in Children: A Moderated-Mediation Model for Inhibitory Control and Gender

**DOI:** 10.3389/fpsyg.2017.01181

**Published:** 2017-07-10

**Authors:** Rosario Cabello, María J. Gutiérrez-Cobo, Pablo Fernández-Berrocal

**Affiliations:** ^1^Department of Developmental and Educational Psychology, University of GranadaGranada, Spain; ^2^Department of Basic Psychology, Faculty of Psychology, University of MálagaMálaga, Spain

**Keywords:** parental education, inhibitory control, aggressive behavior, gender

## Abstract

Aggressive behaviors are highly prevalent in children. Given their negative consequences, it is necessary to look for protective factors that prevent or reduce their progress in early development before they become highly unshakable. With a sample of 147 children, the present study aimed to assess the relation between parental education and inhibitory control in the aggressive behavior of children aged from 7 to 10 years. The participants completed a go/no-go task to assess inhibitory control, whilst their parents reported their education level, and their teachers rated the aggressive behavior of the children through the Teacher Rating Scale (TRS) of the Behavior Assessment System for Children 2 (BASC-2). The results showed that both parental education and inhibitory control determined aggressive behavior in children. In addition, inhibitory control partially mediated the associations between parental education and aggressive behavior after accounting for age. However, a moderated mediation model revealed that lower parental education was associated with higher levels of aggressive behavior, which, in girls occurred independently of inhibitory control. In contrast, inhibitory control mediated this relation in boys. These results suggest the importance of parental education and inhibitory control in the aggressive behavior of children, supporting the idea that both constructs are relevant for understanding these conduct problems in schools, particularly in boys. The clinical implications of these findings are discussed, along with possible future lines of investigation.

## Introduction

The high prevalence of aggressive behavior in children and its negative consequences for individuals and society make it a relevant topic on which to focus (Zych et al., 2015; Calmaestra et al., 2016). When we talk about aggression we are referring to any behavior that is carried out with the intention of causing harm to another individual (Anderson and Bushman, 2002). Both victims of aggressive behaviors and aggressors may demonstrate psychosocial maladjustment, although the victims are those that are harmed the most. While the victims typically show depression, social anxiety, relationship difficulties and loneliness; aggressors report high levels of tension, sleeping problems and hyperactivity (Aluede et al., 2008; Rosen et al., 2009; Evans et al., 2014). In addition, aggressive children may exhibit more serious problems in the future such as antisocial behaviors (Calkins and Keane, 2009).

The General Aggression Model (GAM; DeWall et al., 2011) provides an integrated theoretical explanation for aggressive behavior. According to the GAM, an episode of aggression develops over three stages. The first stage is related to the person (e.g., personality, attachment) and situational inputs (e.g., defiant situation). The second stage refers to internal states (e.g., cognition, feelings) caused by the variables of the first stage. Finally, the last stage is focused on the appraisal and decision-making processes influenced by the second stage, which will lead to the outcome: a pacific or violent response.

Given the prevalence and undesirable consequences of aggression, looking for protective factors that prevent or reduce its progress during childhood is an essential goal. There is already some evidence to suggest that certain variables could be important in reducing these behaviors, such as parental education and the capacity for inhibitory control, both of which have been linked to aggressive behaviors, particularly in adolescents (Raaijmakers et al., 2008; Nocentini et al., 2010; Vuontela et al., 2013; Véronneau et al., 2014; Qiao et al., 2016).

Parental education refers to the level of academic studies achieved by the parents of the children in question. According to the structure of the GAM, parental education belongs to the first stage as a person variable. It has been related to a variety of outcomes such as academic performance, diet, and mental health (Sonego et al., 2013; Kallitsoglou, 2014; Gebremariam et al., 2015). It has also been linked to fewer instances of aggressive behavior in adolescents (Nocentini et al., 2010; Véronneau et al., 2014). For instance, Nocentini et al. (2010) found that a lower level of maternal education was related to higher levels of physical dating aggression. In addition, Véronneau et al. (2014) found that parental education was negatively correlated with problem behaviors such as aggression. However, to our knowledge, this relation in children has not yet been analyzed.

Inhibitory control is the ability to control or stop pre-potent and automatic actions or thoughts, allowing for more flexible and goal-directed behaviors (Miller and Cohen, 2001). This ability is part of the temperamental dimension of effortful control (Rothbart and Bates, 2006), and it plays a very important role in a considerable number of outcomes, exerting its effects over time (Casey et al., 2011). With respect to aggression, lower levels have been found in preschoolers, children, and adolescents with higher inhibitory control (Kochanska and Knaack, 2003; Raaijmakers et al., 2008; Eisenberg et al., 2009, 2010; Hoyle, 2010; Vuontela et al., 2013; Qiao et al., 2016). For instance, Eisenberg et al. (2009) found that inhibitory control clearly predicted externalizing problems such as aggression in elementary school. Inhibitory control operates at the third stage of the GAM as a decision making variable that could favor control over the emission of an aggressive response.

A link has also been found between both of these variables (parental education and inhibitory control). In particular, higher levels of parental education appear to be related to a greater level of inhibitory control (Merz et al., 2014). This relation appears to be very relevant, given the unfeasibility of modifying the parental education variable. Finding a mediating role for inhibitory control in the link between parental education and aggression could open the door to a way of reducing such undesirable conduct in the offspring of less educated parents by, for instance, implementing training programs for inhibitory control. Although some studies have found inhibitory control to be a mediator and moderator variable between outcomes such as aggression and negative emotionality (Suurland et al., 2016), aggression and hostile attribution of intent (Runions and Keating, 2010) or between parental education and early literacy (Merz et al., 2014); to our knowledge, no studies have examined the role of inhibitory control as a mediator of the relation between parental education and aggression in children.

Further, inhibitory control also appears to be influenced by gender. Specifically, higher scores on inhibitory control are usually found in girls (Liu et al., 2013; Merz et al., 2014) from 2 years of age (Gagne and Saudino, 2016). Moreover, when compared with males, females appear to benefit more from inhibitory control training (Mansouri et al., 2016). These gender differences have been explained in terms of biological factors and socialization processes (Else-Quest et al., 2006; Merz et al., 2014). In addition to this, gender differences have also been found in aggression (Anderson and Bushman, 2002).

**FIGURE 1:**
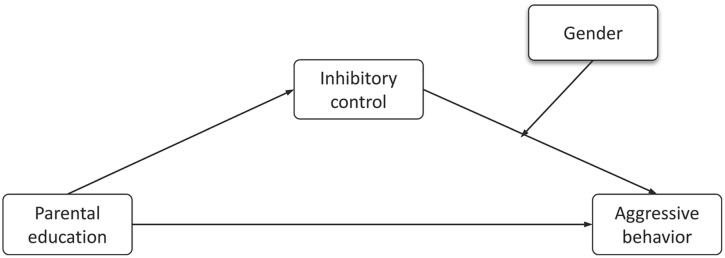
The conceptual moderated mediation model.

On the basis of these considerations, the aim of the present study was to assess the relation between parental education and inhibitory control in the aggressive behavior of children. In particular, we intend to analyze the role of inhibitory control as a mediator in the relation between parental education and aggression, as well as the moderator role of gender in this mediator effect (**Figure 1**). We chose to study these effects in childhood given that this is a critical period for modifying maladjusted behaviors before they become highly unshakable. Given the previous findings reported in the literature, we firstly hypothesized that parental education and inhibitory control would be negatively related to aggression. Secondly, we expect inhibitory control to mediate the relation between parental education and aggressive behavior. Finally, we expect to find a moderator effect of gender when analyzing this mediator role of inhibitory control.

## Materials and Methods

### Participants and Procedure

The sample was composed of 147 children from various Spanish public schools (50% boys), with an age range between 7 and 10 years (*M* = 8.5; *SD* = 1.001). Inclusion criteria were the absence of any (motor or visual) disability or cognitive/language deficits. The parents were informed about this research and no compensation, reward, or incentive was offered in exchange for participation in the study. Of the 155 families who were asked for their consent to participate in the study, 147 (95%) answered positively and completed the questionnaires.

The study was carried out in accordance with the Declaration of Helsinki and ethical guidelines of the American Psychological Association, and all participants (parents and teachers) provided written informed consent. The Research Ethics Committee of the University of Málaga approved the study protocol as part of the projects SEJ-07325 and PSI2012-37490. Trained undergraduate researchers collected information relevant to the study from parents, children, and qualified teachers throughout the course of 1 week. Specifically, the parents provided data on their own levels of education, which could be classified as primary, secondary, or university level. The children were assessed on inhibitory control through the go/no-go task. Finally, the teachers completed the aggressive behavior scale of the Behavior Assessment System for Children (BASC) and the time given to complete it was the same for each teacher.

### Measures

#### Parental Education

Parental education was classified according to the classification system of the Spanish National Institute of Educational Evaluation (OECD, 2016), which distinguishes three levels of education: 1=primary studies; 2=secondary studies; and 3=university studies. For analyses, the educational level of the father and the mother were added together (Gebremariam et al., 2017). We therefore obtained five levels of educational attainment.

#### Inhibitory Control

The Go/no-go task was employed in order to provide a measure of inhibitory control. Our experimental paradigm consisted of a serial presentation of screen-centered blue and red colored circles for the Go stimuli and green and yellow colored circles for the No-go stimuli. The No-go trials were preceded by sequences of 0, 1, 2, 3, 4, 5 Go trials (Durston et al., 2002). The stimuli were presented for 1000 ms followed by an inter-stimulus interval of 1500 ms on a 15′′ computer screen positioned 60 cm in front of the participants. The participants were instructed to verbally respond as rapidly as possible after a Go stimulus (blue and red circles) appeared by saying either “blue,” when a red circle was presented, and “red” when the circle was blue, thereby creating a habit to respond with the opposite color to that presented. For No-go stimuli (green and yellow circles), participants had to say either “green” for green circles or “yellow” for yellow circles, opposite to the response habit created by the GO trials. Therefore, the inhibitory task was encountered when the no-go stimuli were presented, where a change of response was required. Instead of responding with the opposite color to that presented, the participants had to respond with the color that appeared. The task employed 42 stimuli, 30 (71.5%) of which served as Go stimuli and 12 (28.5%) as No-go stimuli, and they were presented pseudo-randomly. Finally, before the task began, the participants completed a practice phase to familiarize themselves with the task. The number of total errors was defined as the primary measure of inhibitory control in the task.

#### Behavior Assessment System for Children (BASC-2)

The Behavior Assessment System for Children 2 (BASC-2; Reynolds and Kamphaus, 2004), Teacher Rating Scale (TRS), was used to assess aggressive behavior. The BASC-2 is an international, well-validated instrument for the socio-emotional evaluation of children and adolescents (Reynolds and Kamphaus, 2004). The BASC-2 is currently utilized by professionals across a range of mental health and school settings. The TRS uses the four-choice Likert response of Never, Sometimes, Often, and Almost Always and provides composite indexes for externalizing problems, internalizing problems, adaptive skills, school problems, and overall behavioral symptoms. In the current study, we used the aggressive scale that is part of the global dimension of the TRS, i.e., externalizing problems, which comprises hyperactivity, aggression, and conduct problems. The Spanish version we used has been shown to have satisfactory psychometric properties (González et al., 2004). In our sample, the alpha coefficient for the aggressive scale was 0.96.

### Data Analyses

All statistical analyses were carried out using the SPSS package (version 20.0; IBM, United States). Preliminary analyses were conducted to compute descriptive statistics. The analysis of the relation between age, gender, parental education, inhibitory control and aggressive behavior scores was conducted with Pearson’s coefficients and Kendall’s Tau-b. To investigate the validity of parental education and inhibitory control for determining aggressive behavior, we conducted three-step hierarchical regression analyses. We also conducted regression analyses to examine the role of inhibitory control as a mediator of the link between parental education and aggressive behavior, based on the recommendations of Baron and Kenny (1986), while hierarchical regression was conducted to examine a moderated mediation model using the PROCESS tool (Hayes, 2013). To directly test our proposed moderated mediation model (**Figure 1**), we used Model 14 in PROCESS to develop and analyze the gender role in our previous mediational model.

## Results

### Sample Characteristics and Correlations between Key Variables

Descriptive statistics and inter-correlations for the study variables are shown in **Table 1**. For all variables except aggressive behavior, skewness and kurtosis values were within an acceptable range of ±2 (Gravetter and Wallnau, 2014). To attenuate skewness and kurtosis of aggressive behavior, this was transformed by adopting the -1/X_1_ function.

**Table 1:** Descriptive statistics and inter-correlations among the measures.

	*M*	Min.	Max.	*SD*	1	2	3	4
(1) Age	8.55	7	10	1	–			
(2) Gender (0=boys)	0.50	0	1	0.50	0.07	–		
(3) Parental education	4.43	2	6	1.47	0.23^∗∗^	0.01	–	
(4) Inhibitory control	0.94	0.83	1	0.04	-0.17^∗^	0.07	0.22^∗∗^	–
(5) Aggressive behavior	49.70	44.71	107.56	9.82	-0.14	-0.14	-0.31^∗∗^	-0.29^∗∗^

*^∗^*p* < 0.05, ^∗∗^*p* < 0.01.*

Age was negatively correlated with inhibitory control and positively correlated with parental education, whilst gender showed no significant correlation with any key variables of the study. Aggressive behavior correlated negatively with parental education and inhibitory control. Finally, parental education correlated positively with inhibitory control.

### Analyses of Hierarchical Regression

With the aim of examining the validity of parental education and inhibitory control as determinants of aggressive behavior, we conducted a three-step hierarchical regression. The determinant variables were age, gender, parental education, and inhibitory control while the dependent variable was aggressive behavior. We conducted the regression by first entering age and gender into the model, followed by parental education, and finally inhibitory control.

The results of the regression model are shown in **Table 2**. Age and gender were not significant determinants of aggressive behavior. Parental education, added at the second step of the models, proved to be a significant determinant of aggressive behavior (Δ*R*^2^ = 0.08), with higher parental education determining lower aggressive behavior. Inhibitory control, added at the last step of the model, proved to be a significant determinant of aggressive behavior (Δ*R*^2^ = 0.06) over and above the effects of parental education.

**Table 2:** Hierarchical regression to determine aggressive behavior from parental education and inhibitory control.

	Aggressive behavior					
	*B*	*SE*	β	*R^2^*	Δ*R^2^*	*F*(*df*)
**Step 1**				0.04	0.04	2.87 (2,144)
Age	-1.31	0.80	-0.13			
Gender	-2.63	1.60	-0.13			
**Step 2**				0.12^∗∗^	0.08^∗∗^	6.55 (3,143)^∗∗∗^
Age	-0.64	0.79	-0.06			
Gender	-2.68	1.54	-0.14			
Parental						
education	-1.97	0.54	-0.29^∗∗∗^			
**Step 3**				0.18^∗∗^	0.06^∗∗^	7.60 (4,142)^∗∗∗^
Age	-1.21	0.79	-0.12			
Gender	-2.27	1.50	-0.12			
Parental						
education	-1.51	0.54	-0.23^∗∗^			
Inhibitory					
control	-56.73	18.35	-0.25^∗∗^			
Total *R*^2^				0.18^∗∗^		

*^∗∗^*p* < 0.01, ^∗∗∗^*p* < 0.001.*

### Mediation and Moderation Analyses

Previous analyses of regression showed that when simultaneously including parental education and inhibitory control in the model, these variables accounted for 18% of the variance in aggressive behavior when age and gender were controlled. This result — which suggests that both constructs are relevant in determining the aggressive behavior of children — led us to construct a mediation model and moderate mediation model to test the relation among these variables. To do so, we first investigated the hypothesis that inhibitory control can be an important mechanism in the relation between parental education and aggressive behavior in children.

**FIGURE 2:**
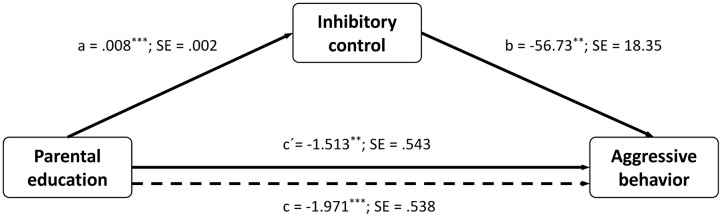
Path coefficients for mediation analysis on children’s aggressive behavior. Dotted lines denote the effect of parental education on aggressive behavior when inhibitory control is not included as a mediator. ^∗^*p* < 0.05, ^∗∗^*p* < 0.01, ^∗∗∗^*p* < 0.001.

In particular, we tested whether inhibitory control mediates the relation between parental education and aggressive behavior. As can be seen in path *c* in **Figure 2**, the mediation analysis showed that aggressive behavior was lower in children whose parents had a higher educational level. Moreover, path *a* in **Figure 2** shows that parental education was positively associated with inhibitory control, suggesting that those children whose parents have a higher parental educational level reported higher inhibitory control. Finally, as can be seen in path *b* in **Figure 2**, inhibitory control was negatively related to aggressive behavior. Using an indirect procedure, the bootstrapped 95% CI confirmed that parental education exerted a significant negative indirect effect on aggressive behavior through inhibitory control (point estimate =-0.46, 95% percentile CI =-1.17 to -0.07). This result points to the possibility that inhibitory control partially mediates the association between parental education and aggressive behavior.

Given that previous research indicates that inhibitory control could be influenced by gender, with higher scores of inhibitory control being reported for girls (Liu et al., 2013; Merz et al., 2014), a moderated mediation model was tested with gender as a possible moderator of the link between inhibitory control and aggressive behavior. **Table 3** presents coefficients, standard errors, and a summary of the moderated mediation model.

**Table 3:** Coefficients, standard error, and summary of the moderated mediation model.

	Criterion				
	Aggressive behavior				
Predictor	Coeff.	*SE*	CI
Inhibitory control	-110.34	24.98	[-159.73, -60.95]
Gender	-101.18	32.32	[-165.08, -37.28]
Inhibitory control ×			
Gender	104.86	34.23	[37.19, 172.54]
Constant	169.22	24.39	[121.00, 217.44]
Summary	*R*^2^ = 0.23		
	*F*(5,141) = 8.31		
	*p* < 0.0001		

Inhibitory control was associated with aggressive behavior, and a gender by group interaction was found for this association (see **Table 3** for coefficients and confidence intervals). Parental education was in turn related to aggressive behavior. When controlling for inhibitory control, the relation between parental education and aggressive behavior was less significant. The 95% bias-corrected bootstrap-CI indicated conditional indirect effects of parental education on aggressive behavior through inhibitory control, although the magnitude of these effects was only significant for boys (**Table 4**). The moderated mediation model accounted for 23% of the variance in aggressive behavior scores.

**Table 4:** Conditional indirect effects of parental education on aggressive behavior through inhibitory control as a function of gender.

	Aggressive behavior	
	Point	95% Bias-corrected bootstrap
Gender	estimate	confidence interval
Boys	-0.89	-2.19 to -0.19
Girls	-0.04	-0.44 to 0.35

## Discussion

The aim of the present study was to examine how parental education and inhibitory control affect aggressive behavior in children. As a further step, we also analyzed whether inhibitory control plays a role in mediating the relation between parental education and aggression, and if gender moderates the mediator role of inhibitory control in the relation between parental education and aggression.

Multiple regression analyses revealed how aggressive behavior in children was determined by both parental education and inhibitory control. In particular, higher scores on each measure appeared to determine less aggressive behavior. These findings are consistent with our first hypothesis and the results of previous studies. For instance, Vuontela et al. (2013) in a sample of children aged between 7 and 12 years, found that lower levels of inhibitory control were related to higher externalizing symptoms, as reported by their parents. Other studies with preschoolers (Raaijmakers et al., 2008; Suurland et al., 2016) have shown that higher levels of aggression were related to lower inhibitory control. These results are also consistent with the findings of other studies in adolescents. For instance, it has been shown that a group of violent adolescents made more errors on a Go/no-go task than a control group (Qiao et al. (2016). Although relatively few studies have evaluated the particular relation between parental education and aggression in children (Letourneau et al., 2013), some studies with an adolescent sample have shown how higher levels of parental education are related to fewer instances of aggressive behavior (Nocentini et al., 2010; Véronneau et al., 2014). Therefore, these two variables are of relevance in terms of reducing aggressive behavior and they operate at different stages of the GAM. In particular, parental education appears to be a protective factor at the beginning of the process as a person input variable while inhibitory control could be beneficial at the third stage of the GAM in the decision making process of the situation.

Interestingly, in our study inhibitory control was found to play a role in mediating, at least in part, the association between parental education and aggressive behavior. Thus, lower levels of parental education are related to higher levels of aggressive behavior through lower inhibitory control, which is consistent with our second hypothesis. Inhibitory control has been demonstrated to be a moderator and mediator variable in a wide range of outcomes (Pardini et al., 2004; Runions and Keating, 2010; Merz et al., 2014; Suurland et al., 2016). However, to the best of our knowledge, this is the first study analyzing this mediation effect. In terms of clinical implications, this mediation effect is of critical importance given the obvious difficulty in modifying the parental education variable. Clearly, modifying parental education is not under our control. Consequently, this mediational effect opens the door to new lines of interventions focused on inhibitory control improvement in these populations with parental education deficits. Future research should therefore aim to investigate whether inhibitory control training could reduce aggressive behavior in children in spite of the lower educational level of their parents. There is already evidence that inhibitory control training can have the effect of reducing aggression in adults, which suggests that this could be a promising field of intervention in children (Denson, 2015).

In our study, inhibitory control was found to partially mediate the relation between parental education and aggression behavior in children. Future research should also focus on looking for additional mediators between parental education and aggression during childhood beyond the inhibitory control that operates at different stages of the GAM. For instance, emotional intelligence (EI) or the ability to perceive, use, understand and regulate emotions (Mayer and Salovey, 1997) may also be a protective factor for children of less educated parents, given its negative correlation with aggressive behavior (García-Sancho et al., 2014) and its positive correlation with inhibitory control (Gutiérrez-Cobo et al., 2017). Specifically, an erroneous and negative perception of our own feelings or those of others could give rise to an unfavorable internal state that results in a negative evaluation of the situation and the subsequent emission of an aggressive response. In contrast, if children learn to adequately perceive their emotions, they are more likely to make a well adjusted evaluation of the situation and suppress the unwanted response (Cabello and Fernández-Berrocal, 2015; García-Sancho et al., 2015). In addition, emotional knowledge is a promoter of prosocial behavior and a protective factor against conduct problems in children of preschool age (Denham et al., 2003). Similarly, children with a lower ability to regulate their emotions could have more problems in controlling the emission of an aggressive response when faced with an emotionally charged situation (Roberton et al., 2012). A further relevant variable that could have a mediational effect between parental education and aggression is empathy. Findlay et al. (2006) showed that children with higher empathy displayed less aggression and more prosocial behaviors than low empathic children. Thus, new areas of research could focus on EI and empathy, given the apparent benefits of training on these variables (Ruiz-Aranda et al., 2012; Nathanson et al., 2016; Castillo-Gualda et al., 2017).

We also found that the mediation effect was moderated by gender. Thus, in our study gender emerged as a moderator of the link between inhibitory control and aggressive behavior, which is consistent with our third hypothesis. Specifically, for the girls in our study, lower parental education was a determinant of aggressive behavior independently of inhibitory control, whilst for the boys inhibitory control played a mediatory role in this relation. Our results are consistent with those previously reported in the literature (Liu et al., 2013), along with the assertion that gender is one of the individual factors that predicts differences in aggression (Anderson and Bushman, 2002). These gender differences also hint at potentially novel areas of investigation since no clear consensus has yet been reached regarding the specific relations between gender and aggression (Brody and Hall, 2014). For instance, there is currently no information with respect to which variables could mediate the relation between parental education and aggressive behavior in girls of this age. Thus, it could be interesting to search for alternative mechanisms underlying the link between parental education and aggressive behavior in girls, which could, for instance, involve socialization processes (Merz et al., 2014). Indeed, social models are usually different for girls and boys, shaping their behaviors in a gender-specific way (Brody and Hall, 2014). Further research, particularly using longitudinal studies, is needed to examine the influence of these processes.

In addition to all of the the results reported here, it is important to note that our sample was composed of children, on which there are considerably fewer studies and where prevention is easier than in older individuals. Indeed, childhood represents an ideal period for training and modifying undesirable behaviors. Moreover, in our study we avoided relying on the subjective perceptions of the children by using external reports for assessing aggressive behavior. Finally, inhibitory control was also measured through an objective laboratory task. Taken together, these aspects of our methodology strengthen the validity of our findings.

## Conclusion

These findings demonstrate that both parental education and inhibitory control are determinants of aggressive behavior in children aged between 7 and 10 years, supporting the idea that both factors are critical for understanding these behavior problems. Furthermore, inhibitory control played a role in mediating the relation between parental education and aggression, but only for the boys in our sample. Future studies are needed to replicate these findings and to further explore the effects of gender on the links between parental education, inhibitory control, and aggression.

## Author Contributions

RC conceived of the study, participated in the data collection, analyzed the data, led preparation and wrote the first draft of the manuscript. MG-C wrote the first draft of the manuscript. PF-B conceived of the study, analyzed the data and contributed to writing the manuscript. All authors contributed to the interpretation of data, helped to draft and revise the manuscript and have read and approved the final manuscript.

## Conflict of Interest Statement

The authors declare that the research was conducted in the absence of any commercial or financial relationships that could be construed as a potential conflict of interest. The reviewer EC and handling Editor declared their shared affiliation, and the handling Editor states that the process nevertheless met the standards of a fair and objective review.
